# Immunogenic cell death-based cancer vaccines: promising prospect in cancer therapy

**DOI:** 10.3389/fimmu.2024.1389173

**Published:** 2024-04-29

**Authors:** Jiandong Wang, Jinyuan Ma, Fangyuan Xie, Fengze Miao, Lei lv, Yueying Huang, Xinyue Zhang, Junxia Yu, Zongguang Tai, Quangang Zhu, Leilei Bao

**Affiliations:** ^1^ School of Pharmacy, Bengbu Medical College, Bengbu, Anhui, China; ^2^ Department of Pharmacy, Third Affiliated Hospital of Naval Medical University, Shanghai, China; ^3^ Shanghai Skin Disease Hospital, School of Medicine, Tongji University, Shanghai, China; ^4^ Shanghai Engineering Research Center of External Chinese Medicine, Shanghai, China

**Keywords:** immunogenic cell death, cancer vaccine, dying tumor cells, immunotherapy, nanoinducers

## Abstract

Tumor immunotherapy is a promising approach for addressing the limitations of conventional tumor treatments, such as chemotherapy and radiotherapy, which often have side effects and fail to prevent recurrence and metastasis. However, the effectiveness and sustainability of immune activation in tumor immunotherapy remain challenging. Tumor immunogenic cell death, characterized by the release of immunogenic substances, damage associated molecular patterns (DAMPs), and tumor associated antigens, from dying tumor cells (DTCs), offers a potential solution. By enhancing the immunogenicity of DTCs through the inclusion of more immunogenic antigens and stimulating factors, immunogenic cell death (ICD) based cancer vaccines can be developed as a powerful tool for immunotherapy. Integrating ICD nanoinducers into conventional treatments like chemotherapy, photodynamic therapy, photothermal therapy, sonodynamic therapy, and radiotherapy presents a novel strategy to enhance treatment efficacy and potentially improve patient outcomes. Preclinical research has identified numerous potential ICD inducers. However, effectively translating these findings into clinically relevant applications remains a critical challenge. This review aims to contribute to this endeavor by providing valuable insights into the *in vitro* preparation of ICD-based cancer vaccines. We explored established tools for ICD induction, followed by an exploration of personalized ICD induction strategies and vaccine designs. By sharing this knowledge, we hope to stimulate further development and advancement in the field of ICD-based cancer vaccines.

## 1 Introduction

Cancer remains the most formidable disease globally, with over 19,292,789 new cases and approximately 9,958,133 cancer-related deaths recorded worldwide each year (1). As is well-known, tumor cells primarily evade immune surveillance by downregulating tumor-associated antigens (TAAs) and tumor-specific antigens (TSAs), and releasing soluble antigens and MHC molecules (2). Therefore, tumor immunotherapy has emerged as a crucial therapeutic approach for suppressing both primary and metastatic tumors. Moreover, immunotherapy can confer long-term immune protection for the body (3). Immune checkpoint blockade (ICB) represents a novel groundbreaking tumor immunotherapy that targets two key immune checkpoint pathways programmed cell death protein 1/programmed death ligand 1 (PD-1/PD-L1) and cytotoxic T lymphocyte-associated protein 4/B7 (CTLA-4/B7). By disrupting the mechanisms of tumor immune resistance, ICB can effectively promote antigen-specific T-cell immune responses. Currently, more than ten ICB drugs have been approved by the FDA for the treatment of a broad spectrum of tumors, offering new hope in the fight against cancer (4). However, the efficacy of ICB drugs appears to be less than satisfactory against late-stage patients in relevant clinical trials, with only a few patients benefiting from these treatments (5).

**Figure 1:**
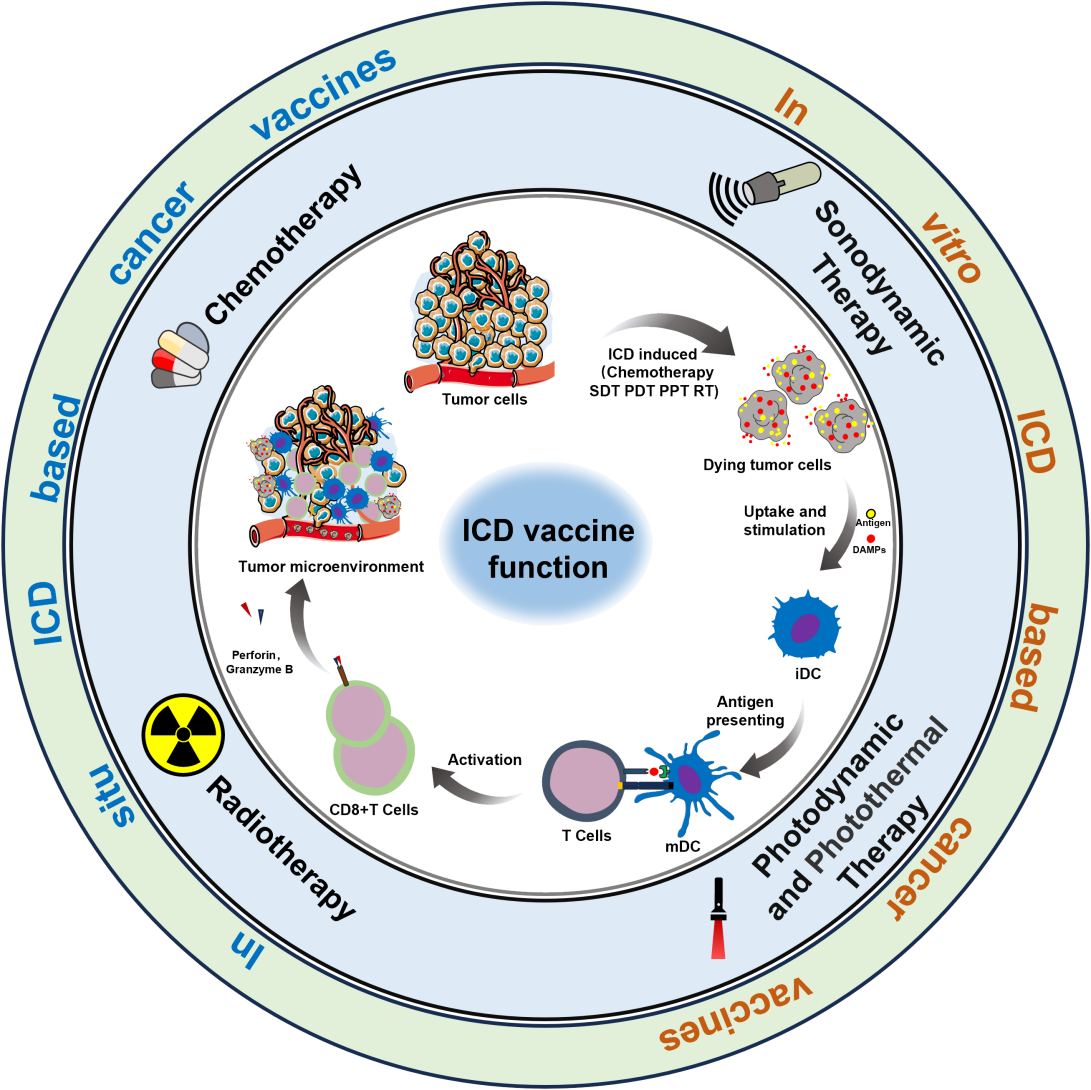
ICD-based cancer vaccines utilize methods to induce anti-cancer immunity within the TME.

Cancer vaccines represent a promising emerging approach in tumor immunotherapy, offering extensive application prospects (6). The complexity of the preparation process, coupled with insufficient antigenic immune effects and immune cell dysfunction, have emerged as the primary factors limiting the development of cancer vaccines (7, 8). Nonetheless, the identification of immunogenic cell death (ICD) as a distinct form of tumor-regulated cell demise has offered new prospects to overcome the limitations hampering the advancement of cancer vaccines (9). In this regard, the capacity of chemotherapy, radiotherapy, photothermal therapy, and sonodynamic therapy to induce ICD, as evidenced in recent studies, could present a novel approach for reshaping conventional methods in oncology (10). It is now understood that the importance of ICD-based cancer vaccines lies in the *in vivo* application of ICD nanomedicine inducers and the production of dying tumor cells (DTCs) *in vitro* during tumor treatment (**Figure 1**).

ICD, induced by the chronic release and exposure of damage-associated molecular patterns (DAMPs) including calreticulin (CRT), adenosine triphosphate (ATP), high mobility histone 1 (HMGB1), heat shock protein (HSP) activates the recruitment and activation functions of neutrophils, macrophages, and dendritic cells. This orchestrated immune response associated within the tumor microenvironment exhibits characteristics akin to vaccination, a concept gaining significant traction within the research community (11). Simultaneously, during the study of ICD induction against tumor cells *in vitro*, it has been discovered that a substantial number of immunogenic DTCs are generated following ICD induction. These DTCs possess a strong ability to release immunogenic substances, such as DAMPs, which continuously trigger a potent immune stress response, remodel the immune microenvironment, and enhance the body’s immune surveillance capabilities. In summary, “ICD-based cancer vaccines” offer advantages such as broad-spectrum antigens and diverse induction conditions, which can mitigate numerous adverse factors in the development and application of cancer vaccines. Overall, the advent of ICD-based cancer vaccines has demonstrated significant potential for the immunotherapy of relevant tumors (12).

However, the pursuit of efficient and stable methods for ICD induction remains critical for developing and evaluating relevant ICD-stimulating nanomedicines. Since ICD inducers and induction strategies are key focuses in ICD-based cancer vaccine research, this review highlights recent advancements in ICD-based cancer vaccines from both *in vivo* and *in vitro* perspectives. Our goal is to provide more reliable preparation protocols and strategies for ICD-based cancer vaccines, thereby promoting their continued development and application. A crucial area of investigation for these vaccines, compared to other immunotherapies, lies in their potential to address tumor recurrence in long-term survivors of metastatic and invasive cancers To achieve curative potential, we advocate for the synergistic combination of immune-stimulating tools with ICD-based cancer vaccine therapy.

## 2 The role of ICD in anti-cancer immunity

Well-documented by numerous studies, immunogenic cell death plays a critical role in generating an immunogenic tumor phenotype, effectively overcoming immunosuppressive effects of a non-immunoreactive tumor microenvironment (TME).ICD is characterized by CRT exposure, ATP release, and leakage of HMGB1 and HSP (13). These processes facilitate the uptake of TAAs by adaptive immune cells, triggering a broad-spectrum antigen-specific immune response, promoting DC maturation, and enhancing the search for DTCs (14). Furthermore, ICB therapy relies on the production and activation of tumor antigen-specific T cells. Consequently, the release of specific antigens from ICD tumor cells is crucial for reshaping the TME and has been validated in the combination of ICB with chemotherapy and radiotherapy. The recruitment of adaptive immune cells, neutrophils, macrophages, and NK cells all can activate innate effector mechanisms (15).

Immunogenic substances released during ICD can be classified into constitutive DAMPs (cDAMPs) and inducible DAMPs (iDAMPs). cDAMPs consist of immune-stimulating molecules such as CRT, ATP, HMGB1, and HSP, which are expressed prior to tumor cell death (16). iDAMPs are endogenous molecules produced by underlying mechanisms during tumor cell death, primarily including cytotoxic T-lymphocytes (CTLs) with CD3+, CD4+, and CD8+, releasing interferon-α (IFN-α), granzymes, lysins, and perforins (17). In this respect, CRT and HSP emit eat-me signals. CRT-CD91 and HSP90-CD91 interactions promote endocytosis signals in tumor cells, inducing antigen presentation and specific CTL responses. Additionally, the release of tumor necrosis factor-α (TNF-α) and interleukin-6 (IL-6) (18); ATP emit energy signals, ATP-P2RY2 binding is involved in the recruitment of monocytes or macrophages, neutrophils, and promoting DC maturation (19); HMGB1, aided by chemokines, binds to pattern recognition receptor (PRR) (P2RX7, P2RY2), CD91, CD40, and Toll-like receptor 4 (TLR4) receptors on the surface of antigen-presenting cells (APCs) (20). Upon the functional activation of APCs, those APCs exposed to TAA and TSA immunostimulants initiate cross-presentation to CD4+/CD8+ T cells, enhancing DC antigen presentation and CTLs proliferation (14). Consequently, the transition from a “cold” to a “hot” tumor immune microenvironment occurs, accompanied by changes in the secretion levels of immunostimulatory and immunosuppressive factors (up-regulation of IFN-γ, TNF-α, and IL-12; down-regulation of IL-4, IL-6, and IL-10), as well as the depletion of myeloid-derived suppressor cells (MDSCs), regulatory T cells (Tregs), and tumor-associated macrophages 2 (TAM2) (21). The above studies overlap in their assertion that ICD, as an in-situ vaccine, possesses potential immunomodulatory abilities and holds great value in reversing the TME and improving the efficacy of tumor immunotherapy.

## 3 Inducers of *in situ* ICD-based cancer vaccines

**Figure 2:**
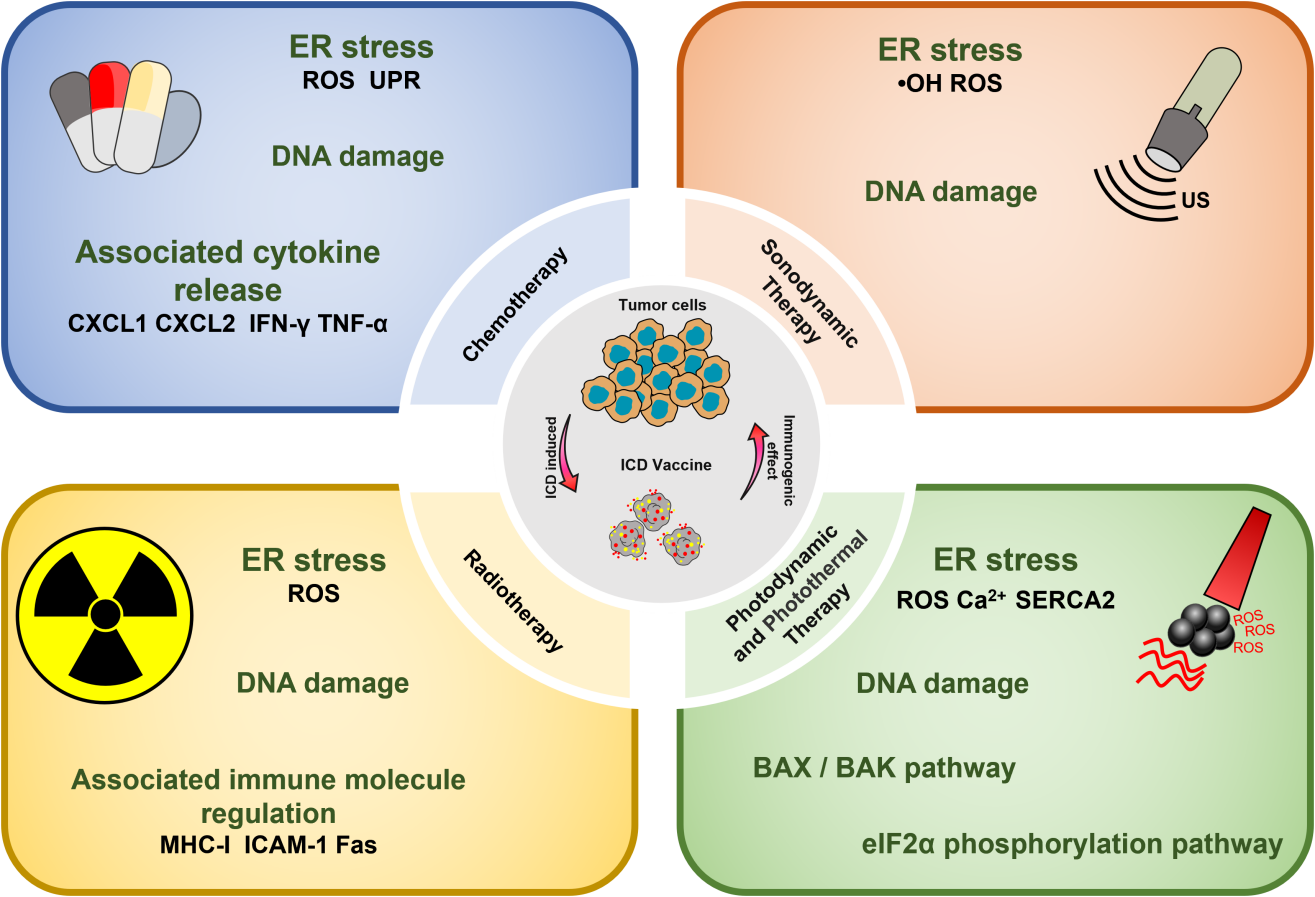
Overview of ICD-based cancer vaccines and the associated mechanisms involved in ICD regulation.

Over the years, a multitude of ICD inducers have been developed, especially when integrated with nanotechnology, to boost the effectiveness of ICDs. Administering ICD nano-inducers to the tumor site not only averts degradation and premature active ingredient release but also enhances their penetration and retention capabilities (EPR) (22). Presently, the primary driving methods of ICDs encompass chemotherapy, photodynamic, photothermal, radiotherapy, and sonodynamic therapies **Table 1**. Thus, a comprehensive investigation of ICD induction mechanisms and inducers holds substantial importance in advancing the development of “*in situ* ICD-based cancer vaccines”. The release of cancer cell immunogenic antigens induced by ICD and the presentation of these antigens by APCs constitute the fundamental stages of the “cancer vaccine-like function” observed *in vivo* (**Figure 2**). We introduce the ICD induction methods with some latest research to provide more evidence in the field of ICD-vaccine.

**Table 1:** ICD-based cancer vaccines with common inducers.

ICD-induced methods	Inducer	Inducer structural formula	Detection of ICD based biomarkers	Ref.
Chemotherapy	Doxorubicin	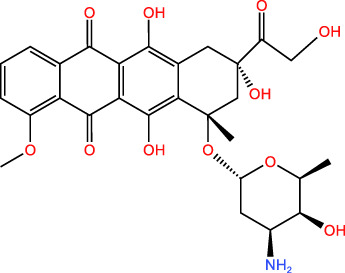	CRT, HMGB1, ATP	(23, 24)
	Oxaliplatin	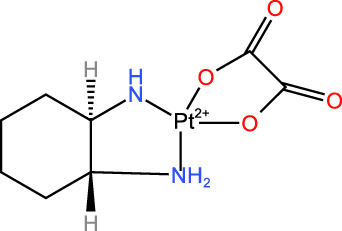	HMGB1	(25, 26)
	Paclitaxel	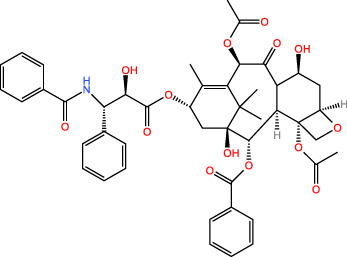	CRT	(27, 28)
	5-Fluorouracil	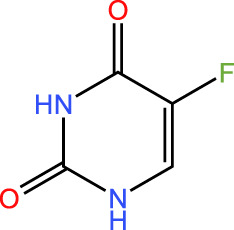	CRT, ATP, HMGB1	(29)
	KP-1339	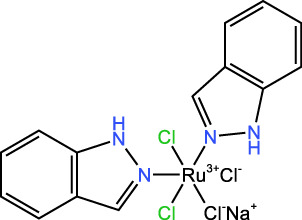	CRT, ATP, HMGB1	(30)
PDT	Chlorine a6	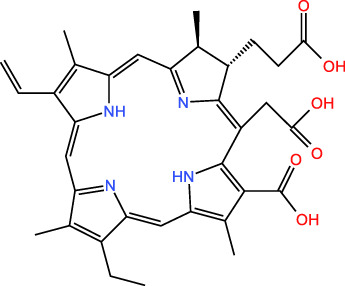	CRT, ATP, HMGB1	(31, 32)
	Hypericin	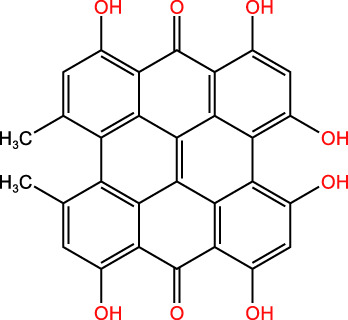	CRT, ATP, HMGB1	(33)
	5-ALA	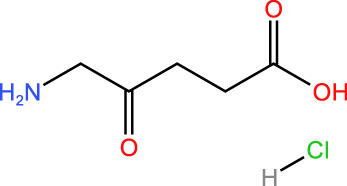	CRT, ATP, HMGB1, HSP	(34, 35)
	Rose Bengal	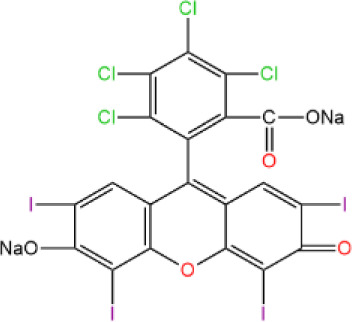	CRT, ATP, HMGB1, HSP	(36)
PPT	ICG	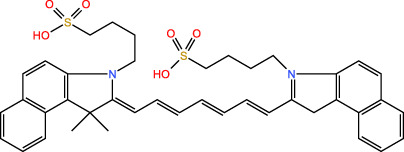	CRT	(37)
	PDA	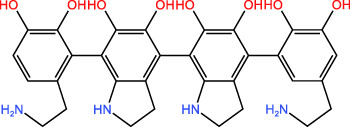	CRT, ATP, HMGB1	(38)
	IR780	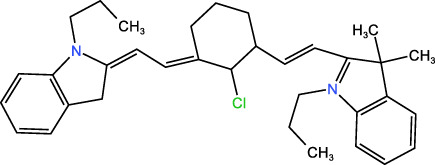	CRT, ATP, HMGB1	(39)
	CuS	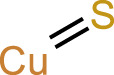	CRT, ATP, HMGB1	(40)
	ZnO	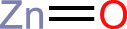	CRT, ATP, HMGB1	(41)
SDT	IR780	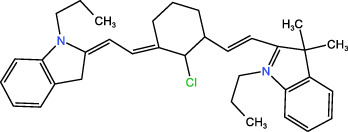	CRT, ATP, HMGB1	(42, 43)
	Perfluorocarbon	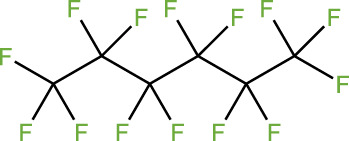	CRT, HMGB1	(44)
	ICG	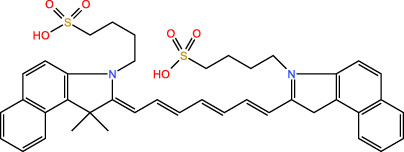	CRT, ATP, HMGB1	(38)
Others	CUR	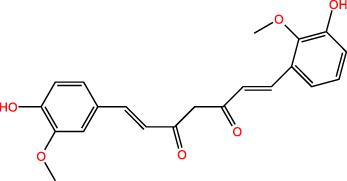	CRT, ATP, HMGB1	(45)
	Icaritin	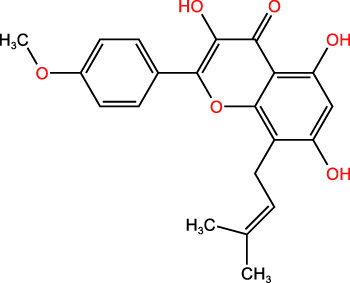	CRT, ATP, HMGB1	(46)

### 3.1 Chemotherapy-induced ICD-based cancer vaccines

Chemotherapeutic agents that have been shown to be effective for ICD induction include: Idarubicin; Epirubicin, Doxorubicin (DOX) (23), Mitoxantrone, Oxaliplatin (Oxp) (25), Bortezomib, Cyclophosphamide, and Paclitaxel (PTX) (27, 47). Studies have demonstrated that the induction of ICD by chemotherapeutic agents, particularly anthracyclines, is accompanied by phenomena such as the unfolded protein response within the endoplasmic reticulum (ER) and the generation of reactive oxygen species (ROS). These events are primarily caused by the DNA damage induced by chemotherapeutic agents to secondary structures, including the cytoplasm (28). Chemotherapy-induced ICD can also lead to the release of CRT, ATP, HMGB1, CXCL1, and CXCL2, which ultimately induces an immune stress response, triggering a sustained antitumor effect (48).

In experiments exploring the use of chemotherapeutic nanomedicines for ICD induction, some researchers employed nanoprecipitation technology to develop and design nanomedicines (called Nano-Folox), which contain Oxp derivatives and FnA. Nano-Folox not only induced ICD, but also synergistically interacted with free 5-Fu to induce a shift from cold to hot tumors. This ultimately led to a significant inhibition of tumor growth in CRC mouse models (29). Liu et al. synthesized liposomes carrying Oxp and coupled with indoximod (IND) precursors; the nanoparticles not only enhanced the ability of Oxp to induce ICD in pancreatic ductal carcinoma (PDAC) but also stimulated antitumor immune responses in PDAC (49). In recent years, researchers have designed nanoplatforms integrating OXA with polyethylene glycolated photosensitizer (PS) prodrugs, exhibiting good stability in blood circulationand able to complete drug release and ICD induction under near-infrared (NIR) irradiation. They also explored the enhancing effect of CD47 blockade on tumor ICD induction (50). Xie et al. designed nanoparticles MDP NPs self-assembled from DOX, MnO2 nanoparticles, Fe3+, and PEG-polyphenol ligands, which could enhance DOX-based tumor ICD induction and achieve high expression of TAAs, DC maturation, and infiltration of tumor-specific T-cells (51). Some researchers have constructed ROS-responsive polymers (R-SIP) using hydrophilic polyethylene glycol (PEG) and a hydrophobic self-immolative backbone, loaded with DOX. This nanoparticle could release DOX in response to spontaneous depolymerization of ROS and undergo depolymerization to produce azoquinone methyl ether derivatives that significantly deplete GSH, increase the level of oxidative stress, and ultimately enhance the induction of ICD in oncological treatments (52).

### 3.2 PDT-induced ICD-based cancer vaccines

PDT has shown effectiveness in inducing tumor cell death, solidifying its role as a viable clinical application in oncology. Currently, photosensitizers with the ability to induce cell death include Hypericin (33), 5-ALA (34), and Rose-Bengal acetate (36). The induction of ICD in tumor cells during PDT treatment is primarily attributed to the generation of intracellular ROS under NIR irradiation, which occurs after photosensitizer aggregation in the ER, causing disruptions in ER homeostasis, elevating calcium levels in the ER, and losing SERCA2 function. Mitochondrial dysfunction, characterized by oxidative damage to mtDNA and BAX/BAK-mediated apoptosis, triggers a series of events culminating in immunogenic stress. This process begins with the release of ATP and exposure of CRT on the mitochondrial surface. Subsequently, DAMPs, such as heat shock protein 70 (HSP70), are released, initiating a localized inflammatory response. Ultimately, these events contribute to the development of an immunogenic stress response (53). Beyond the intrinsic properties of the photosensitizer, the ROS generation and ICD-inducing effects can be severely limited by the constraints of hypoxia and insufficient tumor penetration in the TME (54). Therefore, it is crucial to address adverse factors in the TME to enhance ICD induction by PDT. Several strategies can be employed for this purpose, such as inducing mitochondrial damage that causes ER disorders and calcium overload (55); utilizing oxygen carriers like hemoglobin (Hb) as catalysts for ROS generation, and modifying the targeting effect of ER-targeting pardaxin (FAL) to enable more drugs to be internalized into the ER, thus increasing the efficacy of PDT (37).

Studies onPDT-induced ICD have explored the use of a smart semiconductor polymer nano-immunomodulator (SPNI) that could be activated under acidic TME. When it was used in the therapeutic process, SPNI exerted a photodynamic effect, directly ablating tumors and inducing ICD when exposed to NIR light treatment, while R837 promoted DC maturation and pro-inflammatory cytokine secretion (56). Qiu et al. incorporated the photosensitizer Chlorin e6 (Ce6) doped with the chemotherapeutic agent 10-hydroxycamptothecin (HCPT) into calcified nanocarriers CHC NPs. CHC NPs can generate ROS, causing mitochondrial dysfunction and inducing the ICD. This phenomenon is also crucial to compensate for the lack of results from insufficient immunogenic tumor microenvironment (ITME) in HCPT treatment (31). Zhu et al. demonstrated that a platelet membrane fusion liposome nanovesicle system (named TFL) loaded with type I AIE photosensitizer TBP-2. In this study, TBP-2 has the potential to increase the incidence of cuproptosis and induce the vaccine-function in tumor site (57, 58).

### 3.3 PTT-induced ICD-based cancer vaccines

PTT is a potential non-invasive treatment strategy that converts NIR energy into heat by photothermal agents, ultimately leading to tumor ablation. Interestingly, it was found that PTT could induce ICD and assume a role akin to a vaccine (59). The types of photothermal agents that can induce the photothermal effect at present include (1) precious metals Au, Ag, Pt, etc., which have high photothermal conversion efficiency and imaging; (2) Carbon materials graphene, carbon nanorods, with large photothermal conversion area but poor NIR absorption; (3) Metal and non-metal compounds, CuS, ZnS; with high photothermal and low cost; (4) Organic and inorganic nanomaterials. The ICD-inducing ability of PTT therapy may be attributed to several factors. Firstly, the photothermal agent initiates temperature changes at the tumor site, leading to a Fenton-like reaction, peroxidation reaction, and direct induction of H_2_O_2_ production in the TME (60). These results in intracellular Ca^2+^ overload within tumor organelles (61). The process involves mitochondria damage including reduced membrane potential and the generation of mitochondrial reactive oxygen species (mtROS) and the up-regulation of the PERK-mediated eukaryotic initiation factor 2α (eIF2α) phosphorylation pathway. Disruption of the ER structure is a hallmark of ICD. This process ultimately culminates in the release of a significant amount of DAMPs into the cytoplasm (62).

In recent years, the catalytic properties of Fenton metals, particularly copper and iron, have emerged as crucial mechanisms for inducing cell death processes termed copper death and iron death. The utilization of photo-activated copper, exhibiting exceptional photo-oxidation and reduction catalytic efficiency has ushered in a new era of photosensitizers. This advancement has significantly contributed to the burgeoning research in innovative photodynamic and photothermal therapies for tumors (63). One approach involves the synthesis of multifunctional nanoplatforms, such as Cu-PDA-FA, by combining polydopamine (PDA) with Cu^2+^ through chelation technology. Cu-PDA-FA not only induces ICD and cancer vaccine-like effects but also amplifies the efficiency of conversion (38). Some researchers designed CaO_2_ and Cu_2_Se conjugates, which showed the ability to induce ICD after being activated by NIR-II. Indeed, the Ca^2+^ overload in the ER enhances the immune activation capacity. Reinforcing this concept, research on photothermal materials (40). Zinc oxide (ZnO) has been identified as an efficient drug carrier responsive to tumor pH that significantly inhibits tumor growth. Building on these findings, scientists have designed a multifunctional composite nanoplatform (AuNP@mSiO2@DOX-ZnO) to harness the synergistic therapeutic effects. This platform could promote ICD, maturation of DCs, and proliferation of effector T cells, ultimately preventing tumor growth and metastasis (41). Ran et al. designed nano-platforms PBDB-T NPs using the organic photovoltaic material PBDB-T through a nanoprecipitation method. PBDB-T NPs exhibited favorable photothermal therapeutic effects and the ability to induce ICD. Importantly, they increased the efficiency of DAMPs production after mild-temperature PTT (mPTT) treatment (64). Tian et al. developed a mesoporous polydopamine nanoparticle MPDA. IR-780@MPDA not only induced ICD-activated CTLs in a therapeutic 4T1-homozygous mouse model under NIR treatment, but it also demonstrated its utility for *in vivo* photoacoustic (PA) imaging, as evidenced by the PA imaging tracings (39).

### 3.4 SDT-induced ICD-based cancer vaccines

It is well-established that SDT enables the concentration of ultrasound energy at the tumor site, leveraging the cavitation effect to elevate local temperatures and enhance drug decomposition. This process can generate free radicals and produce ROS under the action of endogenous substances in the cell, thereby achieving the purpose of local killing of the tumor. SDT offers several advantages, inducing high tissue penetration, low invasiveness, high controllability, and low costs (65). Consequently, SDT is safer than the traditional means of tumor treatment, such as chemotherapy, radiotherapy, and can minimize damage to normal tissues during the treatment process. Furthermore, ultrasound (US) has an ideal depth of tissue penetration (10 cm), which greatly mitigates the inhibitory effects of hypoxia, low pH, and other unfavorable factors in complex TME, which confers SDT a stronger ICD induction effect (66).

In recent years, a new type of calreticulin nanoparticles (CRT-NP) has been developed, which can be activated by focused ultrasound (FUS) and induce ICD during melanoma immunotherapy. The CRT-NP *in vivo* therapeutic study of CRT-NP benefits from the non-invasiveness of has the advantage of ultrasound, couple with a thermal effect that transforms the TME to enhance TAA release, HSP expression, and up-regulation of CRT to stimulate tumor immune stress response (67). Some researchers designed perfluorocarbon nanoparticles (LIP-PFH NPs), which also exhibited tumor suppression effect and ICD induction effect on breast cancer cells by SDT (68). Besides, researchers have synthesized mitochondria-targeted liposome nanoparticles (MLipRIR NPs), which could be activated by ultrasound and released R162 that disrupted the glutaminolysis pathway in mitochondria and down-regulates glutathione peroxidase (GPx) enzyme expression. At the same time, IR780 generated large amounts of ROS in response to US treatment, disrupting normal mitochondrial function and inducing ICD (42). Building on the discovery that a combined photodynamic and sonodynamic therapy(PSDT) reduces sonosensitizer dose and energy loss, Zheng et al. developed the OIX-NP. This nanoparticle comprises poly(lactic-co-glycolic acid) (PLGA) encapsulating oxygen-carrying perfluoropentane (PFP), an ICG near-infrared dye and an Oxp chemoinducer. The OIX-NP not only efficiently induce ICD, but also demonstrated significant potential for imaging applications (44).

### 3.5 RT-induced ICD-based cancer vaccines

It has been reported that RT can accumulate energy in the tumor site by using high-energy X-rays, Y-rays, or other isoelectronic radiation (IR). Then RT induces DNA damage, destroying double-stranded DNA (dsDNA), leading to ICD. This process results in the release of key signaling molecules, including CRT, HSP70 and HMGB1, within the TME (69). The induction of ICD by RT mainly involves the up-regulation of histocompatibility complex I (MHC-I) molecule, intercellular adhesion molecule-1, and factor-related apoptosis (Fas) (70). This process contributes to the so-called “distant effect” and inhibits the development of metastasis. Thus, RT has the potential to act as an in-situ vaccine (71, 72).

While radiotherapy is a mainstay of local tumor treatment, its effectiveness in controlling distant tumor spread is limited. However, the recent discovery of RT’s ability to induce ICD offers a promising avenue to revitalize RT therapy. Importantly, research suggests that the degree of immune modulation within the tumor microenvironment by RT is dependent on the radiation dose (73). He et al. found that gold nanoparticles AuNPs were able to increase the expression of phosphorylated eIF2α (p-eIF2α) in G422 glioblastoma cells under RT treatment, promote eIF2α protein phosphorylation, and induce ICD. The study also verified the ICD-inducing ability of AuNPs under RT treatment in a therapeutic G422 tumor-bearing mice model (74).

## 4 *In vitro* induction of DTCs for cancer vaccines

**Figure 3:**
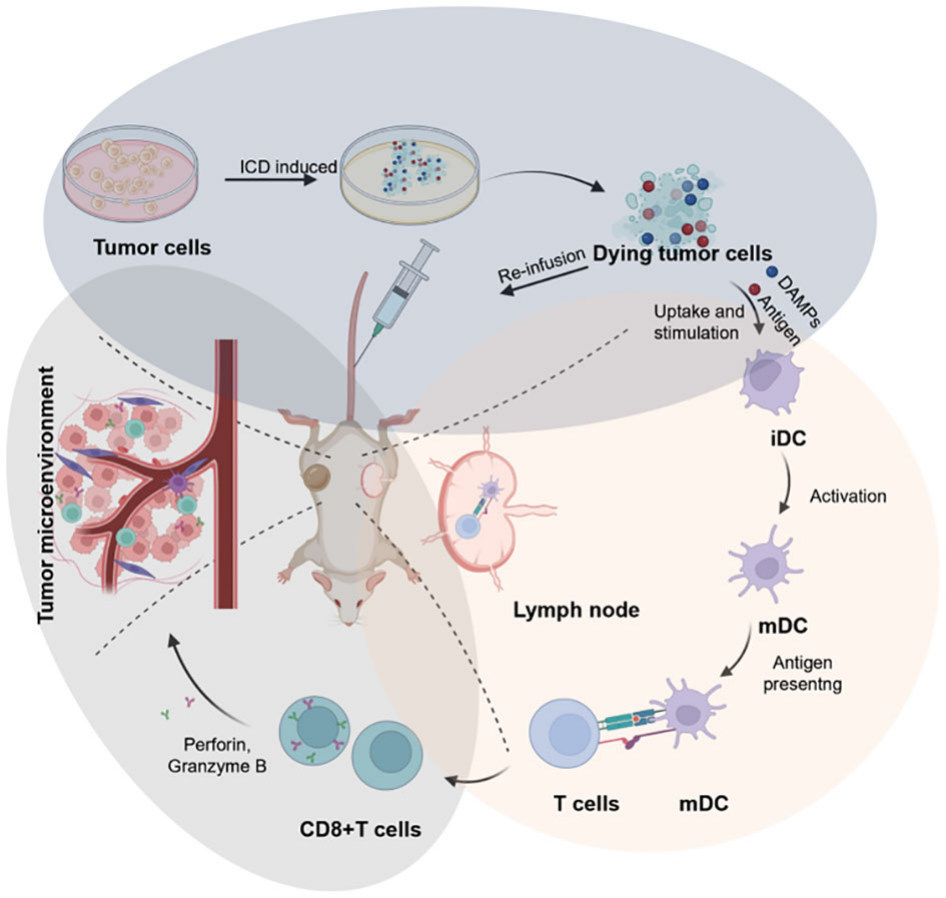
Schematic illustration of the design of cancer vaccines inspired by DTCs for eliciting humoral and cellular immunity, which can be broken into three key stages: the preparation of ICD-based cancer vaccines (blue circle), the immune activation include DC maturation and antigen presentation (red circle), and the immune effect of CD8+T cells in TME for anti-tumor therapy (grey circle). Created by ^©^ 2023 BioRender.

“ICD-based cancer vaccines” involve DTCs for re-infusion vaccination *in vivo*, ultimately leading to tumor therapy. DTCs, produced after treatment with ICD inducers, exhibit excellent immunogenicity (**Figure 3**). In this respect, studies have reported that ICD-based cancer vaccines can induce robust immune activation in mouse models during prophylactic vaccination trials *in vivo* (75). ICD-based cancer vaccines offer a distinct advantage by inducing a comprehensive tumor cell antigen expression profile. This eliminates the dependence on a single antibody for recognition, a major hurdle in conventional immunotherapy. By promoting the expression of diverse tumor antigens, ICD vaccines significantly reduce the risk of tumor cells evading immune attack during treatment. The rationale behind this approach lies in the ability of ICD inducers to stimulate the spontaneous production of relevant antigens, DAMPs, and immune activation molecules within tumor cells. This comprehensive immune response translates to potent antitumor effects, establishment of long-term immune memory against the tumor, and aligns perfectly with the evolving paradigm of immunotherapy, which seeks to integrate preventive and therapeutic strategies.

Studies have explored various methods for the preparation of ICD-based cancer vaccines include: One approach utilizes chemotherapy. Qing et al. demonstrated that DOX can induce tumor cells to become dendritic cell activators. They employed liquid nitrogen cryogenics to generate frozen dying tumor cells (FDTs), which achieved a 38% tumor elimination rate in the MC100 peritoneal carcinoma mouse model. Furthermore, in combination with cytokines IL-12 and aPD-L1, they achieved 100% eradication in the peritoneal metastasis model of colorectal carcinoma (76). Li et al. showed that tumor antigens CIAs induced by chemotherapeutic agents *in vitro*, triggering an immune response and demonstrating synergistic effects with anti-PD-1 therapy (77). Another approach involves using radiotherapy. Researchers have successfully prepared nano-vaccines *in vitro* using RT-induced tumor cells. These vaccines demonstrated efficacy in treating metastatic tumors and enhanced immunotherapeutic effects when combined with anti-PD-1 treatment (78).Although research on ICD inducers and strategies is more advanced, the development of ICD-based cancer vaccines *in vitro* is still in the preliminary stage. However, achieving high efficiency and low cost remains crucial research criteria for ICD-based cancer vaccines. Therefore, investigating whether photodynamic and sonodynamic therapy can be an ideal induction for ICD-based cancer vaccines is expected to be a worthy direction for more researchers to explore. Given the promising results of ICD induction using chemotherapy and radiotherapy, investigating the potential of PDT and SDT as triggers for ICD-based cancer vaccines warrants further exploration. we posit that the personalized design of these ICD-based cancer vaccines holds immense potential for advancing clinical translation in antitumor immunotherapy translation. As an example, previously mentioned strategies like encapsulating patient-specific nanoparticles and obtaining DC activators from tumors offer valuable avenues for personalization. While current personalized cancer vaccine development primarily focus on immune cells loaded with immune-stimulating agents, future breakthroughs lie in utilizing specific immune elements to create novel and highly effective therapeutic strategies.

## 5 ICD related clinical cancer therapy

In clinical immunotherapy studies involving non-small cell lung cancer, hepatocellular carcinoma, breast cancer, bladder cancer, melanoma, squamous carcinoma, and other solid tumors, the induction of ICD has demonstrated the ability to enhance the presentation function of APCs such as DCs, deplete Treg cells, and activate the vitality of immune cytotoxic effector cells. Thess combined effects ultimately lead to improved efficacy in tumor therapy.

As summarized in (**Table 2**), clinical trials have primarily focused on chemotherapy and radiotherapy for inducing ICD, demonstrating its clinical applicability. Researchers have further shown that physical treatments, like cryoablation, can also trigger ICD, potentially improving cost-effectiveness and clinical translation efficiency. However, significant progress is needed to develop and translate ICD-based cancer vaccines for clinical use. Therefore, exploring novel therapeutic approaches based on ICD-based cancer vaccines research and establishing a system to comprehensively evaluate the types and levels of immunogenic substance generated by ICD are crucial next steps.

**Table 2:** Clinical studies about tumor treatment based on ICD.

ICD-based treatment form	Tumor type	Intervention	Aims of the study	Identifier
**Combination of chemotherapy**	Ovarian Cancer	Carboplatin-pegylated Liposomal Doxorubicin (PLD) or Doxorubicin Combination Chemotherapy with Tocilizumab and Pegylated Interferon Alpha (Peg-Intron)	Feasibility of the Combination of Chemotherapy (Carbo/Caelyx or Carbo/Doxorubicin) With Tocilizumab (mAb IL-6R) and Peg-Intron in Patients With Recurrent Ovarian Cancer	NCT01637532
	Hepatocellular carcinoma	Envafolimab; Lenvatinib combined with TACE PD-L1 inhibitor	Envafolimab, Lenvatinib Combined With TACE in the Treatment of Unresectable Locally Advanced Hepatocellular Carcinoma	NCT05582109
	Head and Neck Cancer	Digoxin	Potentiation of Cisplatin-based Chemotherapy by Digoxin in Advanced Unresectable Head and Neck Cancer Patients	NCT02906800
	Colorectal Cancer Metastatic	Capecitabine; Oxaliplatin; Bevacizumab; Pembrolizumab	Chemotherapy and Immunotherapy as Treatment for MSS Metastatic Colorectal Cancer With High Immune Infiltrate	NCT04262687
	Cholangiocarcinoma	Novel combination of chemotherapy and immunotherapy	Durvalumab and Tremelimumab With Platinum-based Chemotherapy in Intrahepatic Cholangiocarcinoma	NCT04989218
	Non-small Cell Lung Cancer	Nivolumab; Oxaliplatin; Ipilimumab	Nivolumab and Ipilimumab in CombinationWith Immunogenic Chemotherapy for Patients With Advanced NSCLC	NCT04043195
	Rectal Neoplasms	Oxaliplatin; Capecitabine and Anti-PD-1 monoclonal antibody	Rectal Artery Infusion Chemotherapy Combined With Anti-PD1 Antibody for MSS LARC	NCT05307198
	Colorectal Cancer Metastatic	Nivolumab FLOX	METIMMOX: Colorectal Cancer METastasis - Shaping Anti-tumor IMMunity by OXaliplatin	NCT03388190
	Non-small Cell Lung Cancer	Atezolizumab and Vinorelbine	Trial to Evaluate Safety and Efficacy of Vinorelbine With Metronomic Administration in Combination With Atezolizumab as Second-line Treatment for Patients With Stage IV Non-small Cell Lung Cancer	NCT03801304
	Solid Tumor	SQZ-AAC-HPV; Ipilimumab; Nivolumab	Study of SQZ-AAC-HPV in Patients With HPV16+ Recurrent, Locally Advanced or Metastatic Solid Tumors	NCT04892043
	Mycosis Fungoides	Cemiplimab	BIOmarker-guided Study to Evaluate the Efficacy and Safety of cemipLimab for advanced Cutaneous T-cell Lymphoma	NCT05538988
	Squamous Cell Carcinoma of the Head and Neck	Atezolizumab and UCPVax	Combination of UCPVax Vaccine and Atezolizumab for the Treatment of Human Papillomavirus Positive Cancers	NCT03946358
	Solid Tumor	RAPA-201 Rapamycin Resistant T Cells and Chemotherapy Prior to RAPA-201 Therapy	RAPA-201 Therapy of Solid Tumors	NCT05144698
	Melanoma	Ipilimumab and Nivolumab	Isolated Hepatic Perfusion in Combination With Ipilimumab and Nivolumab in Patients With Uveal Melanoma Metastases	NCT04463368
	Hepatocellular Carcinoma	Nivolumab and SIR-Spheres	A Study of the Safety and Antitumoral Efficacy of Nivolumab After SIRT for the Treatment of Patients With HCC	NCT03380130
	Ovarian Breast SCLC Gastric Cancers	Olaparib; MEDI4736; Bevacizumab	A Phase I/II Study of MEDI4736 in Combination With Olaparib in Patients With Advanced Solid Tumors	NCT02734004
**Combination of Radiotherapy**	Non- Small Cell Lung Cancer	High-dose radiotherapy alone or concurrent cisplatin-doublet therapy	Detection of Circulating Biomarkers of Immunogenic Cell Death	NCT02921854
	Non-small Cell Lung Cancer	Radiotherapy and atezolizumab/tiragolumab	Study of Stereotactic Ablative Radiotherapy Followed by Atezolizumab/Tiragolumab in Treatment-naive Patients With Metastatic Non-small Cell Lung Cancer	NCT05034055
	Advanced Solid Tumors	Stereotactic Body Radiotherapy; navoximod and NLG802 (indoximod Prodrug)	Safety of Navoximod and NLG802 With Stereotactic Body Radiotherapy Treatment of Advanced Solid Tumors	NCT05469490
	Esophageal Squamous Cell Carcinoma	Radiotherapy combined with immune checkpoint inhibitors	Hybrid Dose-fraction Radiotherapy for Metastatic Non-small Cell Lung Cancer	NCT05348668
	Urinary Bladder Neoplasms	radiotherapy and Tislelizumab	Comprehensive Bladder Preservation Therapy on Patients With Muscle Invasive Bladder Cancer	NCT05445648
	Biliary Tract Neoplasms; Liver Cancer; Hepatocellular Carcinoma	Durvalumab and Tremelimumab combined with Trans-arterial Catheter Chemoembolization Radiofrequency Ablation Cryoablation	A Pilot Study of Combined Immune Checkpoint Inhibition in Combination With Ablative Therapies in Subjects With Hepatocellular Carcinoma or Biliary Tract Carcinomas	NCT02821754
	Melanoma	Radiotherapy and lpilimumab	Trial of SBRT With Concurrent Ipilimumab in Metastatic Melanoma	NCT02406183
**Others**	Breast Cancer	Cryoablation	To Detect Cryoimmunologic Response Induced by Early Breast Cancer Ultrasound-guided Cryoablation	NCT05727813
	Esophageal Cancer	Cryotherapy	Cryotherapy for Locally Advanced Esophageal Cancer	NCT04248582
	Bladder Cancer	Mitomycin C	To Detect Immunogenic Cell Death as a Novel Mechanism of Mitomycin C Activity in Bladder Cancer	NCT04256616
	Thymic Epithelial Tumor; Recurrent Thymoma Thymic Cancer	PT-112	PT-112 in Subjects With Thymoma and Thymic Carcinoma	NCT05104736

## 6 Discussion and conclusion

The immunological adjuvant effect of ICD is now understood to be intricately linked to the exposure and release of cellular DAMPs. These DAMPs include exposed CRT, secreted ATP, ANXA1, TNF-α, and HMGB1, as well as phosphorylated eIF2α. However, additional valuable markers likely remain undiscovered. For example, low levels of autophagy can both protect cells and limit the release of immunogenic substances, while potentially increasing the risk of oncogenicity during treatment. Mounting evidence suggests that tumor autophagy, once thought to be similar to apoptosis, can also impact the effectiveness of antitumor immunotherapy. As expected, the autophagy inducer called STF-62247 (STF) can effectively convert protective autophagy into ICD, thereby enhancing antitumor immune activation. Furthermore, the uncertainty surrounding conventional methods of inducing ICD and determining the optimal dosage of ICD inducers makes it challenging to accurately quantify the release of associated immunogenic DAMPs. As ICD-based cancer therapy gains wider acceptance in clinical settings, it becomes increasingly important to select closely correlated assay secretions for screening ICD inducers that possess efficient induction capabilities for future development of ICD-based cancer vaccines.

Meanwhile, the development of safer and more reliable *in vitro* systems for ICD-based cancer vaccines, as well as the elimination of unfavorable factors *in vivo*, are areas that warrant attention and in-depth exploration. While some personalized ICD-based cancer vaccine research has been discussed above, providing potential mechanisms for their effectiveness, the activation of immunity by cancer vaccines is a complex process. For instance, studies have demonstrated that targeting the STING pathway can activate innate immune signaling in immune-infiltrating cells and within tumor cells, ultimately inducing ICD. However, the effectiveness of STING pathway activation within tumor cells may vary depending on the tumor type., Nanotechnology offers a potential solution by enhancing the delivery of STING activators to cells, thereby improving their efficacy (79). The involvement of the CXCL12/CXCR4 signaling pathway also plays a role in various physiological processes, such as tumor survival, invasion, metastasis, angiogenesis, and the creation of hypoxic environments (80). Additionally, CXCR4 expression can facilitate the transportation of MDSCs in different tumors, leading to the creation of an immunosuppressive TME and immune resistance (81). In gliomas, blocking CXCR4 signaling using nanoparticles loaded with CXCR4 inhibitors can reduce the infiltration of immunosuppressive MDSC and trigger an adaptive immune response (82).

In recent years, immunotherapy has made remarkable strides in the treatment of cancer, particularly with the emergence of cancer vaccines based on immune cell death. These vaccines hold great promise for expanding and enhancing tumor immunotherapy. In this article, we expounded on the mechanisms of ICD as a potent tool for regulating TME and explored the vast potential of ICD-based cancer vaccines. The successful clinical application of ICD-based cancer vaccines necessitates a rigorous evaluation of their therapeutic value. Therefore, we advocate for the identification of more robust clinical evaluation indicators, particularly those that assess the efficacy of ICD induction by these vaccines. A comprehensive assessment of both safety and efficacy is paramount in determining the transformative potential of this approach in cancer treatment. Further exploration of the intricate mechanisms underlying ICD and its role in tumor immunotherapy is crucial to unlocking the full potential of this innovative strategy. Our ongoing research endeavors to contribute to the expanding body of knowledge surrounding ICD-based cancer vaccines and ultimately pave the way for their successful translation into clinical practice through meticulous evaluation and analysis. In conclusion, the utilization of immunogenic patterns generated by ICD in tumor cells represents a paradigm shift in the field of tumor immunotherapy. By advancing the development of disease-specific cancer vaccines, we can harness the full potential of this approach to significantly improve treatment outcomes for cancer patients. Rigorous evaluation and analysis are essential to ensure the clinical relevance and applicability of these vaccines, ultimately leading to improved patient outcomes on a global scale.

## Author contributions

JW: Software, Visualization, Writing – original draft, Writing – review & editing. JM: Investigation, Supervision, Writing – review & editing. FX: Supervision, Writing – review & editing. FM: Supervision, Writing – review & editing. LL: Supervision, Writing – review & editing. YH: Investigation, Writing – review & editing. XZ: Supervision, Writing – review & editing. JY: Supervision, Writing – review & editing. ZT: Supervision, Writing – review & editing. QZ: Funding acquisition, Writing – review & editing. LB: Funding acquisition, Supervision, Writing – review & editing.

## Glossary

**Table:**

DAMPs	damage-associated molecular patterns
DTCs	dying tumor cells
ICD	immunogenic cell death
TAAs	tumor-associated antigens
TSAs	tumor-specific antigens
ICB	Immune checkpoint blockade
PD-1/PD-L1	programmed cell death protein 1/programmed death ligand 1
CTLA-4/B7	cytotoxic T lymphocyte-associated protein 4/B7
CRT	calreticulin
ATP	adenosine triphosphate
HMGB1	high mobility histone 1
HSP	heat shock protein
cDAMPs	constitutive DAMPs
iDAMPs	inducible DAMPs
TME	tumor microenvironment
CTLs	cytotoxic T-lymphocytes
IFN-α	interferon-α
TNF-α	tumor necrosis factor-α
IL-6	interleukin-6
PRR	pattern recognition receptor
TLR4	Toll-like receptor 4
APCs	antigen-presenting cells
MDSCs	myeloid-derived suppressor cells
Tregs	regulatory T cells
TAM2	tumor-associated macrophages 2
EPR	enhances penetration and retention
DOX	Doxorubicin
Oxp	Oxaliplatin
PTX	Paclitaxel
IND	indoximod
PDAC	pancreatic ductal carcinoma
PS	photosensitizer
R-SIP	ROS-responsive polymers
PEG	polyethylene glycol
ER	endoplasmic reticulum
ROS	reactive oxygen species
GSH	glutathione
NIR	near-infrared
Cas9-Cdk5	CRISPR/Cas9-Cdk5 plasmid
HSP70	heat shock protein 70
Hb	hemoglobin
FAL	ER-targeting pardaxin
SPNI	semiconductor polymer nano-immunomodulator
Ce6	Chlorin e6
HCPT	hydroxycamptothecin
ITME	insufficient immunogenic tumor microenvironment
mtROS	mitochondrial reactive oxygen species
TLR7	Toll-like receptor 7
poly (ethylene glycol)-block	poly(2-(diisopropylamino)ethyl methacrylate)
PBEs	phenylboronic acid esters
PPa	photosensitizer pheophorbide a
aPD-L1	anti-PD-L1 antibody
eIF2α	eukaryotic initiation factor 2α
PDA	polydopamine
ZnO	Zinc oxide
PA	photoacoustic
BSA	bovine serum albumin
mPTT	mild-temperature PTT
US	ultrasound
CRT-NP	calreticulin nanoparticles
FUS	focused ultrasound
LIP-PFH NPs	perfluorocarbon nanoparticles
MLipRIR NPs	mitochondria-targeted liposome nanoparticles
GPx	glutathione peroxidase
PFH	perfluoro hexane
EOC	epithelial ovarian cancer
PSDT	PDT/SDT treatment modality
PLGA	poly (lactic-co-glycolic acid)
PFP	perfluoropentane
IR	isoelectronic radiation
dsDNA	destroying double-stranded DNA
MHC-I	mainly involves the up-regulation of histocompatibility complex I
Fas	factor-related apoptosis
H@Gd-NCPs	Hemin@ Gd3+/5′ -GMP NCPs
5′-GMP	5′-guanosine monophosphate
Gd-NCPs	Gd3+/5′-GMP NCPs
p-eIF2α	phosphorylated eIF2α
FDTs	frozen dying tumor cells
LNs	lymph nodes
PLD	Carboplatin-pegylated Liposomal Doxorubicin
Peg-Intron	Pegylated Interferon Alpha
SBRT	Stereotactic Body Radiotherapy
TACE	Trans-arterial Catheter Chemoembolization
RFA	Radiofrequency Ablation
STF	STF-62247
